# Systematic Review of the Use of Intravenous Ketamine for Fibromyalgia

**DOI:** 10.31486/toj.21.0038

**Published:** 2021

**Authors:** Mila Pastrak, Alaa Abd-Elsayed, Frederick Ma, Bruce Vrooman, Ognjen Visnjevac

**Affiliations:** ^1^ Faculty of Health Sciences, McMaster University*,* Hamilton, Ontario, Canada; ^2^ Department of Anesthesiology, University of Wisconsin School of Medicine and Public Health, Madison, WI; ^3^ Bloor Pain Specialists, Toronto, Ontario, Canada; ^4^ Department of Anesthesiology and Pain Management, Dartmouth-Hitchcock Medical Center, Lebanon, NH; ^5^ Department of Anesthesiology and Pain Management Cleveland Clinic Canada, Toronto, Ontario, Canada

**Keywords:** *Chronic pain*, *fibromyalgia*, *infusion–intravenous*, *ketamine*

## Abstract

**Background:** Fibromyalgia, a complex disorder that affects 1% to 5% of the population, presents as widespread chronic musculoskeletal pain without physical or laboratory signs of any specific pathologic process. The mechanism, while still being explored, suggests central sensitization and disordered pain regulation at the spinal cord and supraspinal levels, with a resulting imbalance between excitation and inhibition that may alter central nervous system nociceptive processing. Nociceptive hypersensitivity results from activity of the N-methyl-D-aspartate receptor (NMDAR)–mediated glutamatergic synaptic transmission in the spinal cord and brain. Because ketamine, an NMDAR antagonist, may reduce induction of synaptic plasticity and maintenance of chronic pain states, the study of its use in intravenous form to treat fibromyalgia has increased.

**Methods:** We conducted a literature search with the objectives of examining the effect of intravenous ketamine administration on pain relief, identifying side effects, and highlighting the need for clinical studies to evaluate ketamine infusion treatment protocols for patients with fibromyalgia. We used the keywords “fibromyalgia,” “chronic pain,” “ketamine,” “intravenous,” and “infusion” and found 7 publications that included 118 patients with fibromyalgia who met inclusion criteria.

**Results:** Clinical studies revealed a short-term reduction—only for a few hours after the infusions—in self-reported pain intensity with single, low-dose, intravenous ketamine infusions, likely attributable to nociception-dependent central sensitization in fibromyalgia via NMDAR blockade. Case studies suggest that increases in the total dose of ketamine and longer, more frequent infusions may be associated with more effective pain relief and longer-lasting analgesia. Another neurotransmitter release may be contributing to this outcome.

**Conclusion:** This systematic review suggests a dose response, indicating potential efficacy of intravenous ketamine in the treatment of fibromyalgia.

## INTRODUCTION

Fibromyalgia is a complex disorder that affects 1% to 5% of the population and can occur at any age.^1^ Fibromyalgia presents as a widespread chronic musculoskeletal pain without physical or laboratory signs of any specific pathologic process. Most patients also experience fatigue, memory problems, sleep disturbance, depression, and anxiety.^2^ Similar to other chronic pain disorders, prevalence rates of fibromyalgia are higher in females than in males.^3^ The underlying mechanisms for the continued pain in fibromyalgia are still being explored. A substantial amount of research evidence supports that a low threshold to pain is caused by central sensitization and disordered pain regulation at the spinal cord and supraspinal levels.^4,5^ Increased reactivity of pain-processing cells in the spinal cord or brain evokes hyperexcitability of pain pathways combined with decreased activity of inhibitory pathways.^6^ Thus, the imbalance between the amount of excitation and inhibition results in alterations of central nervous system (CNS) nociceptive processing.

An important mechanism at the spinal cord level is the interaction between the peripheral mechanoreceptors and the deep spinal cord neurons. The evidence shows that in chronic pain conditions, peripheral sensory neurons and axons show synaptic remodeling in the spinal dorsal horn, resulting in nociceptive hypersensitivity that is dependent on the activity of the N-methyl-D-aspartate receptor (NMDAR).^7^ As such, low-level non-noxious stimuli are perceived as pain because of the increased sensitivity of deep spinal cord neurons that transmit sensory information and amplified trafficking of pain signals. At the supraspinal level, chronic pain is associated with the changes of sensory regions in the brain responsible for nociceptive processing. Specific brain structures that display greater activation include the secondary somatosensory cortex, insula, and anterior cingulate cortex.^8,9^ Functional neuroimaging studies in patients with fibromyalgia compared to normal individuals showed higher brain activation in the thalamus, primary and secondary somatosensory cortices, insula, and cingulate cortex with pressure, heat, or nociceptive stimuli.^10-13^ Patients with fibromyalgia also showed impairment of the descending inhibitory pain pathways that resulted in decreased activation in the anterior cingulate cortex and thalamus, as well as a significant imbalance in connectivity within the pain network during rest.^14^

The greater activation of some regions and the inhibition of others likely result in changes in the neurotransmitter levels in the brain. A decrease in serotonergic and noradrenergic activities of the inhibitory monoaminergic pain control pathways has been proposed as one of the mechanisms in underlying chronic pain.^15,16^ Further, clinical treatment that simultaneously increases serotonin and norepinephrine levels has been successful in restoring descending inhibitory activity in fibromyalgia.^2^ Altered dopaminergic and GABAergic neurotransmission may also contribute to the augmented central processing of pain in patients with fibromyalgia.^17^ Brain imaging in patients with fibromyalgia has shown elevated glutamate levels in pain-related brain regions such as the posterior cingulate gyrus, posterior insula, ventrolateral prefrontal cortex, and amygdala.^18^ Patients with fibromyalgia also show increased glutamate and glycine in cerebrospinal fluid.^19^ When patients are treated with pregabalin, elevated glutamate levels in the insula and functional connectivity between the default mode network and insula decrease with both clinical and experimental pain.^18^

The pathogenesis of fibromyalgia appears to have a strong genetic component. A genome-wide linkage study confirmed a potential inheritance mechanism by showing a 13.6-fold increased risk of developing the syndrome in first-degree relatives.^20^ Gene research suggests that fibromyalgia is potentially associated with polymorphisms in the serotoninergic, dopaminergic, and catecholaminergic systems, predisposing an individual to an insufficient analgesic response.^21^ Two potential susceptibility candidate genes are the serotonin transporter gene (SLC64A4) and the transient receptor potential vanilloid channel 2 gene (TRPV2) on chromosome 17p11.2–q11.2.^20^ Polymorphism of SLC64A4 and TRPV2 has been linked to endogenous pain modulation in patients with fibromyalgia.^21,22^ Two additional genes are the myelin transcription factor 1 like gene (MYT1L), responsible for neuronal differentiation and cognitive ability, and the neurexin 3 gene (NRXN3), a receptor in the nervous system and a cell adhesion molecule.^23^ However, no single nucleotide polymorphism has achieved the genome-wide significant threshold.

The NMDAR mediates the excitatory glutamatergic synaptic transmission in the spinal cord and brain that is strongly implicated in the amplification of pain signals and central sensitization in fibromyalgia.^7^ Consequently, NMDAR antagonists such as ketamine may be used to reduce the excessive nociceptive input and central pain sensitization. In clinical practice, ketamine [2-(2-chlorophenyl)-2-(methylamino)cyclohexanone] is primarily used for anesthesia and perioperative analgesia. Ketamine acts as a noncompetitive inhibitor that binds an intrachannel NMDAR site, resulting in decreased channel opening time.^24^ In clinical use, the S (+) stereoisomer of ketamine is the most potent form of NMDAR antagonist.^24^ Because ketamine blockade of NMDARs has the potential to reduce the induction of synaptic plasticity in central and peripheral neurons and prevent functional changes responsible for the maintenance of chronic pain states, ketamine's use for chronic pain management has increased since 1995.^25^ Ketamine also interacts with other receptors and channels in the CNS, including monoaminergic receptors, opioid receptors, nicotinic and muscarinic acetylcholine receptors, and voltage-sensitive sodium signaling.^26^ Ketamine binds with high affinity to dopamine D2 receptors. Suppression of the normal activity of dopamine-releasing neurons within the limbic system is hypothesized to contribute to pain in fibromyalgia.^27^ Because dopamine plays a dominant role in natural analgesia, potentiation of dopamine signaling may contribute to ketamine's analgesia. The potential of ketamine to enhance endogenous antinociceptive systems results in potentiation of the descending serotoninergic inhibition pathways.^28^ Further, ketamine may also reduce substance P receptor–mediated pain and restore altered brain-derived neurotrophic factor (BDNF) expression.^25^ Ketamine also has an anti-inflammatory effect, modulating the production of different proinflammatory mediators that are implicated in the pathogenesis of fibromyalgia.^29^ Therefore, in addition to ketamine's effect on NMDAR, functional changes in neuroplasticity in chronic pain may relate to multiple neuronal pathways that are implicated in fibromyalgia pain physiology.

Because the same neurotransmitters that control pain also regulate sleep, cognition, energy, and emotional networks in the brain, these functions/neural networks may improve in patients with fibromyalgia.^30^ Besides having analgesic properties, ketamine is an effective treatment for severe major depressive disorder, a condition that has a high prevalence in patients with fibromyalgia.^31,32^ Based on ketamine's robust antidepressant effect, the US Food and Drug Administration approved a nasal spray formulation in March 2019 for the management of treatment-resistant depression.^33^

The evidence of ketamine's effectiveness in fibromyalgia is evolving. The objectives of this review were to examine the effect of intravenous (IV) ketamine administration on pain relief, to identify side effects, and to highlight the need for clinical studies to evaluate ketamine infusion treatment protocols for patients with fibromyalgia.

## METHODS

We conducted a literature search on ketamine for the treatment of fibromyalgia in PubMed using the following search keywords: “fibromyalgia,” “chronic pain,” “ketamine,” “intravenous,” and “infusion.” We limited the search to human subjects, English language, and articles with full text available. We searched the bibliographic sections of all manuscripts for additional relevant citations. Both prospective and retrospective studies were included, as well as smaller studies and case reports.

### Search Strategy and Study Selection

Although ketamine is used in the management of a number of chronic pain disorders, few studies have evaluated the use of IV ketamine in patients with fibromyalgia. We identified 106 potential articles. Once the duplicate records were removed, we screened the remaining 92 publications and excluded an additional 71 records because the publications primarily focused on other chronic pain syndromes treated with ketamine. Of the 21 full-text articles we assessed, we excluded 10 articles because they did not include patients with a confirmed diagnosis of fibromyalgia and 4 articles because they involved modes of ketamine administration other than IV. The 7 reviewed publications included 4 randomized controlled trials with IV ketamine involving 82 patients (3 trials had a crossover design; 1 trial had a parallel design), 1 prospective trial (34 patients) that did not include a placebo control, and 2 case studies (1 patient each) with IV administration (Figure).^34-40^ A total of 118 patients with fibromyalgia, 106 of whom were treated with ketamine, were included in this systematic review, and data were subjected to qualitative analysis. As summarized in the Table, the heterogeneous design of the studies regarding dose and duration of ketamine infusion, use of various outcome measurements, and concomitant medications between studies prevented quantitative analyses of the data.

**Figure.**
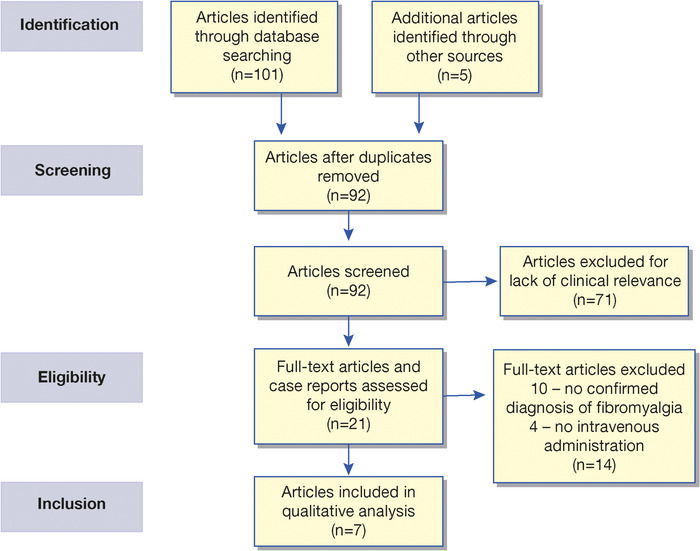
Preferred reporting items for systematic reviews and meta-analyses flow diagram showing the identification and selection of literature for review.

**Table.** Summary of Published Ketamine Infusion Studies

Study	Study Design	Participants	Ketamine Regimen	Results	Side Effects
Sörensen et al, 1995^34^	PRCT; crossover (placebo/ketamine)	11	0.3 mg/kg over 10 minutes	1. Reduced VAS at the end of infusion and 20 to 80 minutes after	Short-lasting (up to 15 minutes) feeling of unreality, dizziness, and changes in hearing
				2. Six of 8 responders had reduction in pain for 2 to 7 days	
Sörensen et al, 1997^35^	PRCT; crossover (placebo/ketamine)	18	0.3 mg/kg over 10 minutes	Physical functioning ability score (Fibromyalgia Impact Questionnaire) improved postinfusion	None
Graven-Nielsen et al, 2000 (Part 1)^36^	PRCT; crossover (placebo/ketamine)	29	0.3 mg/kg over 30 minutes	58% of patients had at least a 50% reduction in VAS	None
Graven-Nielsen et al, 2000 (Part 2)^36^	PRCT; crossover (placebo/ketamine)	15	0.3 mg/kg over 30 minutes	1. No change in VAS duration, reduced VAS pain area, and VAS peak	None
				2. Reduced local pain and referred pain area	
				3. Decreased temporal summation	
				4. No effect on electrical pain threshold	
Noppers et al, 2011^37^	PRCT; parallel (active placebo)	24	0.5 mg/kg or 5 mg midazolam over 30 minutes	1. Reduction in VAS >50% during and at the end of infusion	Mild-to-moderate, short-lasting (approximately 30 minutes postinfusion) drowsiness and euphoria in both the ketamine and midazolam groups
				2. No significant differences in pain reduction between the ketamine and midazolam groups at 2.5 hours, 1 week, or 8 weeks postinfusion	
Cohen et al, 2006^38^	Prospective study	34	0.1 mg/kg over 7 minutes, followed by oral dextromethorphan	1. Reduction in VAS >50% in 18 of 34 patients	Short-lasting (approximately 30 minutes postinfusion) dizziness, confusion, euphoria, and nausea
				2. Significant correlation between pain relief with ketamine and dextromethorphan (*P*<0.001)	
Hanna and Smith, 2016^39^	Case study	1	Day 1, 200 mg (plus 10 mg diazepam, 8 mg ondansetron); day 2, 600 mg (plus 10 mg diazepam, 8 mg ondansetron, 6 mg midazolam); and days 3 to 5, 800 mg	VAS at the start was 7/10, and after third infusion, was 0/10; VAS remained at 0/10 for >1 year	Nausea, agitation
Hanna et al, 2018^40^	Case study (fibromyalgia and rheumatoid arthritis)	1	Day 1, 428 mg; days 2 and 3, 856 mg; and days 4 to 10, 1,063 mg	Before treatment, pain was 10 on an 11-point pain scale; after the 10th infusion, pain score was 0 to 1; 2-day booster infusion was administered 3 weeks later when pain levels returned to 40% of pre-ketamine levels; 3 months after the booster infusion, another 2-day booster infusion was administered with pain levels at 30% to 40% of preketamine levels; 3 months after the booster infusion, pain score was 2	None

PRCT, prospective randomized controlled trial; VAS, visual analog scale.

## RESULTS

In a double-blind, placebo-controlled study by Sörensen et al, 11 female patients with fibromyalgia received a single dose of ketamine infusion (0.3 mg/kg) or placebo (saline) in a crossover design.^34^ A significant reduction (>50%) was seen in pain intensity scores measured by the visual analog scale (VAS) at the end of the infusion (*P*<0.05) and after 20 to 80 minutes in ketamine-treated patients vs placebo (*P*<0.01 and *P*<0.001, respectively). In the majority of ketamine-exposed patients, pain alleviation lasted 2 to 7 days. Patients treated with ketamine also reported a significant decrease in tenderness (*P*<0.02), increased muscle endurance (*P*<0.02), decreased pain threshold (*P*<0.02), and pain tolerance at tender points and control points (*P*<0.02 and *P*<0.03, respectively).^34^

Sörensen et al further tested pain response in 18 patients with fibromyalgia in a randomized, double-blind, crossover study with IV administration of ketamine (0.3 mg/kg), lidocaine, morphine, and saline.^35^ Patients who responded to ketamine showed sustained improvements in pain threshold and pain tolerance for up to 120 minutes relative to placebo and had significantly improved Fibromyalgia Impact Questionnaire (FIQ) scores.

Another randomized, double-blind, crossover study by Graven-Nielsen et al in 29 patients with fibromyalgia investigated IV ketamine at the 0.3 mg/kg dose.^36^ In the first part of the study, patients who showed >50% pain decrease on 2 consecutive VAS assessments were identified as ketamine responders. Of the 17 responders, 15 were exposed to the effect of either placebo or 0.3 mg/kg ketamine in the second part of the study that assessed a central mechanism of muscular nociceptive activity such as muscle pain and temporal summation. When local pain and referred pain were induced by intramuscular infusion of hypertonic saline, patients experienced a progressive reduction of VAS scores with 30-minute ketamine infusions (*P*<0.05), showing attenuation of muscular hyperalgesia and muscle pain at rest. The pain intensity area also showed significant reduction relative to placebo (*P*<0.02). Ketamine infusion also resulted in a decrease of temporal summation after cutaneous and muscular electrical stimulation in comparison to placebo.^36^

Noppers et al examined the robustness of response to a single infusion of S-ketamine at 0.5 mg/kg to an infusion of 5 mg midazolam (active placebo group) in 24 patients with fibromyalgia in a double-blind, placebo-controlled study.^37^ The initial VAS and FIQ scores were assessed 2.5 hours after infusion and weekly for 8 weeks. Similar to the findings of other studies, Noppers et al found that ketamine caused a significant initial pain reduction (*P*<0.01 vs baseline). Although ketamine infusion resulted in a significantly higher number of responders in comparison to midazolam (*P*<0.05), no significant difference in treatment effect on fibromyalgia pain and experimental heat pain was found 2.5 hours after the infusion or during the 8-week follow-up between the ketamine- and midazolam-treated patients.^37^

These 4 double-blind, prospective, randomized controlled trials, which tested low doses of ketamine between 0.3 and 0.5 mg/kg administered for 10 to 30 minutes, showed significant reductions of pain during and at the end of the ketamine infusion. Although sustained pain decreases with ketamine relative to placebo were observed by Sörensen and colleagues for up to 2 hours, the study by Noppers and colleagues did not show a prolonged effect of ketamine action.^34,37^

The study by Cohen et al in 34 patients with fibromyalgia with low-dose ketamine infusion (0.1 mg/kg) followed by oral dextromethorphan treatment showed a VAS reduction of >50% in 18 patients and a significant correlation between pain relief with ketamine and dextromethorphan, but no long-term benefit from the 1-time ketamine infusion was observed.^38^

In these earlier studies, the S-ketamine formulation used by Noppers et al^37^ was similar to the less-potent racemic ketamine in terms of the duration of the clinically useful benefit. Results from this review of low-dose ketamine infusion studies suggest that the duration of infusion is the primary factor associated with clinically meaningful, short-term analgesia and can be linked to the pharmacokinetics of ketamine.

In contrast to these initial studies, recent case studies (2016 and 2018) with IV ketamine for pain treatment of patients with fibromyalgia used higher doses of ketamine and greater frequency and duration of administration. Hanna and Smith administered IV ketamine to a patient with fibromyalgia over 4 hours at doses escalating from 200 mg to 800 mg for 5 days, with a booster of 800 mg 2 weeks later; the result was long-lasting remission from chronic pain and improved quality of life.^39^ The patient was simultaneously treated with diazepam and midazolam to relieve anxiety and promote sedation, as well as ondansetron to control nausea, a common side effect associated with ketamine administration. In another case study by Hanna et al, a patient with fibromyalgia and rheumatoid arthritis received 10 consecutive ketamine infusions with escalating ketamine doses from 428 mg to 1,063 mg.^40^ The patient's pain score gradually decreased during treatment and remained at 80% improvement from the preketamine levels, with booster ketamine infusions administered approximately every 3 months. Taken together, these 2 cases suggest that higher doses and longer, repeated ketamine infusions have the potential to achieve sustained analgesia in patients with fibromyalgia.

Unpleasant mild to moderate psychomimetic side effects were reported with low total doses of ketamine in the above studies. Other common ketamine-related adverse outcomes included nausea, dizziness, confusion, and drowsiness. These side effects were short lasting and resolved within approximately 30 minutes after infusions. On the other hand, the higher doses of ketamine used in the 2 case studies were well tolerated, and no unexpected adverse effects were observed, including hepatotoxicity.

## DISCUSSION

In patients with fibromyalgia, central sensitization is demonstrated as pain hypersensitivity, specifically diffuse allodynia and pressure hyperalgesia.^5,15^ As summarized in the Table, clinical studies in patients with fibromyalgia demonstrate a short-term reduction in self-reported pain intensity with single, low-dose, IV ketamine infusions. Examination of low-dose ketamine studies suggests that ketamine was able to reduce both pain and pressure hyperalgesia, to increase pain tolerance at pressure points, and to affect temporal summation. The observed findings suggest the potential of ketamine to reduce hyperalgesia from nociception-dependent central sensitization in fibromyalgia via NMDAR blockade. However, no single, low-dose, IV ketamine infusion study found significant benefits that lasted longer than the first few hours postinfusion.

Although studies comparing the effectiveness of higher doses of ketamine administered more frequently for longer periods are lacking, the 2 case studies provide a potential direction for future clinical research. The infusion protocols from the case studies suggest that increases in the total dose of ketamine administered, longer duration of infusions, and more frequent infusions are required to produce a higher degree of pain relief and possibly longer-lasting analgesia. Because the scientific evidence supports a major role for central sensitization in the generation of the symptoms of fibromyalgia, prolonged ketamine infusions and higher total doses are more likely to induce NMDAR blockade and activation of the alpha-amino-3-hydroxy-5-methyl-4-isoxazolepropionic acid receptor (AMPAR), resulting in the release of other neurotransmitters, leading to synaptogenesis and changes in neuroplasticity. Based on preliminary clinical data, patients with fibromyalgia may be able to achieve a longer-term analgesic effect with prolonged and repetitive IV ketamine infusion regimens.

The literature about ketamine infusion for other pain conditions shows that patients experience transient psychomimetic side effects such as dysphoria and sedation, regardless of infusion duration or total dose infused.^41^ However, serious side effects that require close monitoring may occur, particularly with higher doses and prolonged ketamine infusions. Ketamine action on a number of receptors, such as NMDAR, acetylcholine, histamine, opioid, and monoamine, may be associated with psychomimetic, gastrointestinal, and cardiovascular adverse consequences; therefore, the need for controlled, multiple-dose studies is necessary.^41^

Clinical evidence shows that the pharmacodynamic actions of ketamine that induce rapid and sustained antidepressant actions overlap with those seen with ketamine administration for fibromyalgia.^42^ The mechanism of action of ketamine as an antidepressant includes direct synaptic or extrasynaptic NMDAR inhibition, AMPAR activation, glutamate surge with enhanced glutamatergic firing, and synaptogenesis, resulting in the reversal of the negative effects of depression.^43,44^ Major depressive disorder is characterized by synaptic downregulation in the prefrontal cortex and hippocampus. Functional magnetic resonance imaging in treatment-resistant depression supports normalization of prefrontal dysconnectivity with ketamine.^44^ Ketamine's antidepressant actions on synaptic plasticity also include enhanced release and translation of BDNF.^45^ Published evidence also points to similarities in neuroendocrine changes, psychological symptoms, and physical symptoms between fibromyalgia and major depression, although the underlying construct between the 2 conditions is not known.^46^ In addition to the role of NMDAR in the pathophysiology of chronic pain and depression, NMDAR is important for learning and memory that are often affected in patients with both conditions. These mechanisms—direct synaptic or extrasynaptic NMDAR inhibition, AMPAR activation, glutamate surge with enhanced glutamatergic firing, synaptogenesis, and neuroplasticity—of ketamine action may also account for enhanced neurocognitive function that is associated with the improvement in depressive symptoms with ketamine.

The ability of ketamine to modulate NMDAR, glutaminergic signaling, BDNF, descending inhibition, and inflammation and thereby result in a reduction of central sensitization may provide a comprehensive approach for the management of patients with fibromyalgia. Overall, ketamine has the potential to provide an alternative avenue for managing diverse CNS alterations that contribute to the symptoms of fibromyalgia.

## CONCLUSION

The growing body of literature suggests that ketamine infusions impact a number of aspects of pain pathology in fibromyalgia. Large prospective, placebo-controlled clinical trials with different infusion protocols and long-term follow-up periods are needed to determine the effectiveness, dose response, and safety of ketamine infusion as a therapeutic modality for fibromyalgia.

## Notes

*This article meets the Accreditation Council for Graduate Medical Education and the American Board of Medical Specialties Maintenance of Certification competencies for Patient Care and Medical Knowledge.*
